# Identification of Antibacterial Peptide Candidates Encrypted in Stress-Related and Metabolic *Saccharomyces cerevisiae* Proteins

**DOI:** 10.3390/ph15020163

**Published:** 2022-01-28

**Authors:** Maria Fernanda da Silva Santos, Cyntia Silva Freitas, Giovani Carlo Verissimo da Costa, Patricia Ribeiro Pereira, Vania Margaret Flosi Paschoalin

**Affiliations:** 1 Chemistry Institute, Federal University of Rio de Janeiro, Avenida Athos da Silveira Ramos 149, Cidade Universitaria, Rio de Janeiro 21941-909, Brazil; santos_fernanda@yahoo.com.br (M.F.d.S.S.); freitas.cs@pos.iq.ufrj.br (C.S.F.); patriciarp@iq.ufrj.br (P.R.P.); 2 Nova Friburgo Health Institute, Fluminense Federal University, Nova Friburgo 28625-650, Brazil; giovaniverissimo@id.uff.br

**Keywords:** baker’s yeast, biomass autolysate, FPLC <10 kDa peptides, nano-LC-MS/MS, in silico screening, food-grade pharmaceuticals, antimicrobial peptides

## Abstract

The protein-rich nature of *Saccharomyces cerevisiae* has led this yeast to the spotlight concerning the search for antimicrobial peptides. Herein, a <10 kDa peptide-rich extract displaying antibacterial activity was obtained through the autolysis of yeast biomass under mild thermal treatment with self-proteolysis by endogenous peptidases. Estimated IC_50_ for the peptide pools obtained by FPLC gel filtration indicated improved antibacterial activities against foodborne bacteria and bacteria of clinical interest. Similarly, the estimated cytotoxicity concentrations against healthy human fibroblasts, alongside selective indices ≥10, indicates the fractions are safe, at least in a mixture format, for human tissues. Nano-LC-MS/MS analysis revealed that the peptides in FPLC fractions could be derived from both induced-proteolysis and proteasome activity in abundant proteins, up-regulated under stress conditions during *S. cerevisiae* biomass manufacturing, including those coded by *TDH1/2/3*, *HSP12*, *SSA1/2*, *ADH1/2*, *CDC19*, *PGK1*, *PPI1*, *PDC1*, and *GMP1*, as well as by other non-abundant proteins. Fifty-eight AMP candidate sequences were predicted following an in silico analysis using four independent algorithms, indicating their possible contribution to the bacterial inactivation observed in the peptides pool, which deserve special attention for further validation of individual functionality. *S. cerevisiae*-biomass peptides, an unconventional but abundant source of pharmaceuticals, may be promissory adjuvants to treat infectious diseases that are poorly sensitive to conventional antibiotics.

## 1. Introduction

*Saccharomyces cerevisiae* cells have been exploited for biotechnological purposes for millennia, particularly due to their ability to ferment carbohydrates found in food matrices or in food derivatives, even when under unfavorable metabolic conditions and/or as part of a complex microbiota consortium, producing highly appreciated beverages such as wine and beer, as well as baked goods [1,2]. In addition, *S. cerevisiae* and its cellular components can introduce different features to fermented food matrices, frequently modifying their flavor and taste but also conferring antioxidant and anti-inflammatory properties to the primary matrices. The Food and Drug Administration (FDA), in title 21 of the Code of Federal Regulations (21 CFR), part 172, has conferred a GRAS (generally recognized as safe) *status* (https://www.ecfr.gov/cgi-bin/ECFR?page=browse, accessed on 20 September 2020) to this microorganism, allowing for the addition of entire cells and/or their derivative compounds to food products, meaning that they can be safely used in human tissues [3].

Based on this, the use of *S. cerevisiae* by the food industry has evolved over the years to include the application of commercial yeast biomass as a pure source of specific yeast lineages refined for different purposes, which has allowed for the standardization of bakery product and beverage manufacturing worldwide [4]. Millions of tons of yeast biomass are produced every year, and part of them are discarded, which has raised scientific interest in searching for alternative uses by applying the functional and innovative concept of circular bio-economy, aggregating economic value to this GRAS sub-product [5,6].

It has been reported that bioactive peptides displaying health-promoting effects both in vitro and in vivo, such as antihypertensive, antioxidant, antimicrobial, immunomodulatory, anti-obesity, anti-diabetes, and mitogenic properties, can be found encrypted in yeast precursor proteins, especially those involved in glucose utilization and abiotic stress tolerance. When hydrolyzed, yeast-derived proteins release various bioactive peptides that can act against microbial pathogens. Although these antimicrobial peptides (AMPs) have not been extensively studied, evidence demonstrates that they exhibit promising pharmacological potential against foodborne and infectious microorganisms [7]. In addition, AMPs are less prone to trigger bacterial resistance mechanisms compared to conventional antibiotics, although AMP-resistant bacteria have been described, albeit at a lower frequency. The low propensity of AMPs in triggering bacterial resistance resides in their multiple intracellular and surface membrane mode of action, targeting stable molecules such as lipid II, lipopolysaccharides, or phospholipids that are not easily mutated. This contrasts from antibiotics, which act on unstable and mutation-sensible molecules. The advantage of AMPs over antibiotics includes their broad spectrum of action, low tendency to elicit resistance mechanisms, ability to stimulate the immune system, and the possibility of molecule modification by engineering strategies to manage their effectiveness [8,9,10,11]. Moreover, most AMPs do not display an affinity to human membrane components, which are rich in zwitterionic lipids and cholesterol, suggesting low or null cytotoxicity, and are thus considered valuable candidates for antibiotics and as chemical food preservative replacements [8,12,13,14].

Because of this, the present study aims to produce and identify antimicrobial peptide candidates derived from baker’s yeast biomass proteins displaying the ability to inactivate clinical and foodborne bacteria. Peptide release will be promoted through a simple, non-toxic, and naturally-induced autolytic and proteolytic process accompanied by fractionation by gel filtration chromatography, followed by an in vitro evaluation of antimicrobial spectra, in vitro toxicological assays against healthy human cell lineages, mass spectrometry identification of the primary peptide sequences in the pool fractions, and in silico screening for the prediction of AMP candidate sequences.

## 2. Results

### 2.1. Characterization and Fractionation of Yeast Extract Peptides

**Figure 1:**
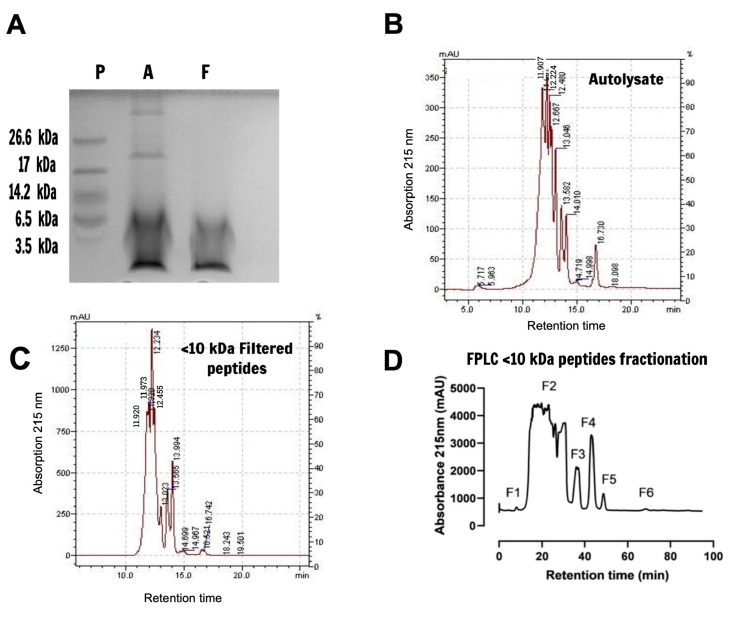
Yeast extract characterization and fractionation. (**A**) Tricine-SDS-PAGE 16% of about 70 μg of the autolysate (lane A), filtered <10 kDa peptide extract (lane F), and ultra-low range molecular markers (lane P). Peptide bands, previously fixed with 5% glutaraldehyde, were visualized after staining with 0.025% Brilliant Blue G. (**B**) Fractionation of the protein/peptide content in the autolysate and (**C**) <10 kDa filtered peptide extract was performed employing an HPLC system coupled to a gel filtration ProSec300S column equilibrated with 0.05 M sodium phosphate buffer pH 7, and eluted in the same buffer containing 0.15 M NaCl at a flow rate of 1 mL/min. (**D**) 14.78 mg of the <10 kDa filtered peptides extract were fractionated using an FPLC Akta purifier system equipped with a Superdex-75/10 300GL column equilibrated and eluted with 0.05 M sodium phosphate buffer pH 7.0 at a constant flow of 0.8 mL/min. Absorbances were monitored at 215 nm, and fractions were pooled to compose fraction 1 to fraction 6 (F1–F6) according to the retention time of each peak.

An autolysate extract was prepared using baker’s yeast biomass as the protein source, and the release of encrypted peptides was conducted by self-proteolysis through the activation of endogenous peptidases by thermal treatment for 24 h. The resulting proteins and peptides resolved by 16% tricine-SDS-PAGE indicate that the autolysate extract is mostly composed of fragments with molecular masses under 10 kDa, although some molecules displaying masses higher than 17 kDa or 26 kDa are also present (Figure 1A, lane A). As expected, after autolysate ultrafiltration through a 10 kDa cutoff membrane, the extract contained only peptides with molecular masses under 10 kDa (Figure 1A, lane F), indicating the suitability of the applied methodology for peptide generation. Minor peaks at 5.2 min retention time were observed in the autolysate chromatogram, (Figure 1B), corresponding to molecular masses above 75 kDa (conalbumin) (Figure S1). Major peaks were detected from 11.9 min to 16.7 min in both the autolysate and <10 kDa filtered peptides extracts, corresponding to <10 kDa fragments that are carried down even under 3.5 kDa, according to the last protein molecular weight marker in the tricine-SDS-PAGE run (Figure 1B,C and Figure S1). This indicates that the high molecular mass contamination observed in the autolysate seems to be eliminated through 10 kDa cutoff membrane ultrafiltration, resulting in a <10 kDa filtered peptides extract containing only small peptides.

The <10 kDa filtered peptides extract was then fractionated by FPLC gel filtration, resulting in six fractions displayed in the chromatogram, as presented in Figure 1D, with retention times varying from 7 to 69 min and peptide concentrations ranging from 7.36 to 752.18 µg/mL.

### 2.2. Antimicrobial Activity Displayed by the Autolysate and <10 kDa Filtered Peptide Extracts and FPLC Gel Filtration Fractions

Although high concentrations are required, both the autolysate and <10 kDa filtered peptides extracts were able to inactivate the growth of Gram-positive and Gram-negative bacteria at different levels. Inhibition curves reached over 50% in most cases, except for *Acinetobacter* genomospecies 3 with the autolysate and <10 kDa filtered peptide extracts and *Escherichia coli* CDC O55 with the <10 kDa filtered peptide extracts. The estimated inhibitory concentration (IC_50_) ranged from 428.5 to 9088 µg/mL for the autolysate and from 547.9 to 9939 µg/mL for the <10 kDa filtered peptides (Table 1). Except for *Aeromonas hydrophila* ATCC 7966, which was inhibited by the autolysate at IC_50_ = 994.2 µg/mL, *Bacillus cereus* ATCC 11778, which was inhibited by the autolysate at IC_50_ = 428.5 µg/mL, and the <10 kDa filtered peptides at IC_50_ = 547.9 µg/mL, all other assayed bacteria were only affected at concentrations over 1.7 mg/mL and were, thus, not considered effective inhibitors. Comparing the antimicrobial efficiency of both extracts, an improvement trend can be observed for the <10 kDa filtered peptides against *Aeromonas hydrophila* ATCC 7966, *Acinetobacter* genomospecies 3, *Escherichia coli* CDC EDL-933, and *Salmonella enterica* ATCC 12325, although no statistically significant differences in the inactivation effect, evaluated through the IC_50_ following autolysate ultrafiltration, were observed. This is an expected behavior as, at this stage, the study was conducted with crude extracts not only from bioactive peptides and proteins, but also from other types of interfering molecules. However, since the <10 kDa filtered peptides are free from high molecular weight peptide contaminants, this extract was chosen for use in the subsequent fractionation step.

The FPLC gel filtration fractions of the <10 kDa filtered peptides extract exhibited significantly increased antimicrobial activity when tested against various bacterial species compared to the <10 kDa peptide filtered extract and the autolysate as indicated by statistical analysis (Table 1 and Figure S2).

Although the antimicrobial performance of the peptides pool was promissory, when compared to the reference antibiotic chloramphenicol, none of the peptide fractions exhibited higher IC_50_ values, which should be an expected result, as the analysis was assayed with a mixture of peptides and not a purified molecule. It is possible that, when assayed alone, some individual peptides would reach concentrations higher than those found in the peptide pool fractions, and then present IC_50_ improvements. Further studies with isolated peptides should be conducted to validate their functionality and understand their individual contributions.

Data monitoring the progress of active peptide fractionation were included in Table S1 by considering the individual estimated IC_50_ values as one antimicrobial unit (AU) for each gel filtration fraction and for the <10 kDa filtered peptide extract. Peptide pool fractions (F1–F6) fractions were included in the fractionation table when estimated IC_50_ values lower than 1000 µg/mL were observed, as estimated IC_50_ values markedly higher than those obtained by conventional antibiotics were disregarded (Table S1). The specific activities and the fractionation fold increased for all fractions, indicating that separation was successfully performed, resulting in the enrichment of the fractions containing antimicrobial peptides and/or the elimination of interfering compounds, leading to improvements of the antimicrobial estimated IC_50_ values for most of the fractions. Low recovery percentages of the peptide pool were obtained from the original source, indicating that future studies with individual sequences could be better achieved with synthetic forms (Table S1).

### 2.3. Toxicological Evaluation of the <10 kDa Filtered Peptide Extract

All samples, the autolysate, the <10 kDa filtered peptides, and the FPLC gel filtration fractions affected the viability of a healthy human fibroblast lineage (HFF-1) in a dose-dependent manner, with high estimated CC_50_ values ranging from 1464 to 4642 μg/mL (Table 2 and Figure S3). Different from the antimicrobial activity, in this case, the autolysate ultrafiltration resulted in a significant improvement in the estimated CC_50_ of the <10 kDa filtered peptides. The same was observed when comparing F1–F3 to the <10 kDa filtered peptides.

Since the cytotoxicity assay was performed with a peptide mixture, the individual contribution of any peptide must be investigated by assaying the synthetic or purified form of the best AMP candidates proposed in this study. The autolysate and the <10 kDa filtered peptide extracts exhibited 50% cytotoxic estimated concentration (CC_50_) levels very close to their respective antimicrobial IC_50_, as demonstrated by their low selective index values (SI ≤ 1), except when assayed against *Bacillus cereus* ATCC 11778, *Aeromonas hydrophila* ATCC 7966, *Shigella sonnei* ATCC 25931, and *Staphylococcus aureus* ATCC 14458 (Table 2). The highest effectiveness of both yeast extracts was achieved against *Bacillus cereus* ATCC 11778, with a selective index of 3.42 for the autolysate and 5.95 for the <10 kDa filtered peptides. Moreover, autolysate ultrafiltration successfully improved the SI values of the <10 kDa filtered peptides for all investigated bacteria, indicating partial cytotoxic compound elimination, an effect more clearly demonstrated following FPLC gel filtration fractionation.

The <10 kDa filtered peptides fractionated by FPLC gel filtration resulted in additional SI value improvements for all FPLC fractions compared to the yeast extracts, indicating the high selectivity of these peptide pools against most of the bacteria strains, accompanied by very low cytotoxicity, with estimated CC_50_ values ranging from 10-fold to 453-fold higher than their estimated antimicrobial IC_50_ values (Table 2 and Figure S3). The estimated results suggest that F1 may exhibit high selectivity against eight bacteria species, except for *Shigella flexneri* ATCC 12022 and *Staphylococcus aureus* ATCC 14458, with SI values ≥ 10 up to 453, indicating that this peptide pool could contain the most promising peptides. Similarly, F4 peptides may exhibit high selectivity against *Aeromonas hydrophila* ATCC 7966, *Bacillus cereus* ATCC 11778, *Escherichia coli* CDC EDL-933, and *Escherichia coli* DH5α, with SI values ranging from 17 to 43. Furthermore, F2 and F3 peptides may selectively inhibit *Aeromonas hydrophila* ATCC 7966, and F5 may selectively inhibit *Escherichia coli* DH5α, since the estimated SI values were higher than 10 (Table 2).

As the selective index, SI, is comprised of a ratio between the cytotoxic concentration (CC_50_) and the effective antimicrobial concentration of peptide pools in yeast biomass extracts or fractions (IC_50_)_,_ some authors consider that SI values should ideally be ≥10. This means that the cytotoxic concentrations of the bioactive compound are 10-fold higher than the IC_50_ and the peptide pool then exhibits non-toxic effects at its effective antimicrobial concentration [16]. On the other hand, it should be stated that their application must be preceded by the determination of their effective antimicrobial concentrations, evaluated by MIC and IC_50_, as well as their cytotoxic concentrations (CC_50_), which should be further confirmed for each individual AMP sequence in their synthetic forms.

### 2.4. Peptide Identification and In Silico Screening for AMP Candidates

Nanoscale liquid chromatography coupled to tandem mass spectrometry (nano-LC-MS/MS) analysis of the peptides in the FPLC gel filtration fractions (F1–F6) revealed an extensive set of 778 peptide hits found in the three replicates with ppm values between −6 and 6, (Table S2). The identified peptides displayed molecular masses ranging from 884 to 4344 Da and originated from proteins encoded by 179 different genes, with the ten most predominant genes being *TDH3*, *HSP12*, *SSA1*, *TDH2*, *PGK1*, *TEF1*/2, *ENO1*, *TDH1*, *ENO2*, and *CDC19*. Many of these identified peptides were distributed in more than one fraction (Table S2).

The in silico analysis of these peptides using four independent algorithms based on Support Vector Machines (SVMs), Random Forests (RF), Discriminant Analysis (DA), and Artificial Neural Network (ANN) models were performed to predict the probability of a peptide sequence displaying potential antimicrobial activity. The candidate sequences were confronted with experimentally validated AMP sequences, previously classified in 45 families categorized according to AMPs conserved sequences signatures, organized in 36 patterns and 78 Hidden Markov Models (HMMs) that contain physicochemical features inherent to an effective AMP. Based on this, the prediction is determined according to different algorithmic strategies that include a classification using an ensemble of trees (RF), pattern recognition using a polynomial function-based model (SVM), classification through a linear combination of independent variables (DA), or a neural network model inspired in the brain neuronal system (ANN), resulting in accuracies of 93.4% (RF), 92.6% (SVM), 87.5% (DA), and 86.9% (ANN) [17,18]. A total of 20 promising AMP sequences were classified as the best, as they tested positive for three or four algorithms. One (Table 3, in bold letters) belongs to the most effective peptide pool, F1. Another 38 peptides were classified as good candidates, as they tested positive for two algorithms. In this regard, seven candidates were found in F1 (Table 3, in bold letters).

## 3. Discussion

The versatility of *Saccharomyces cerevisiae* has been employed for millennia to produce fermented foods such as cheese, bread, and other baked goods, as well as beverages including wine and beer, whose manufacturing has been standardized worldwide by the use of commercial yeast biomass [1,2,4]. Besides its GRAS status, low-fat and low-sodium contents, and its ability to transform food matrices for human consumption, *S. cerevisiae* also provides nutritional and health-promoting compounds, such as amino acids, β-glucans and mannans, lipids, B-complex vitamins, minerals, and oligopeptides, which contribute to the management of diabetic foot ulcers by controlling fungal infections and wound healing, cholesterol levels, allergic symptoms, and microorganism growth [2,3,19,20,21,22,23,24]. Spent brewer yeast or baker’s yeast extracts, rich in oligopeptides, are widely marketed for dietary supplementation purposes, and their biological properties are explored to confirm the claimed bio-functionalities, such as antioxidant, antihypertensive, antimicrobial, alpha-glucosidase inhibitor, anti-lipolysis, anti-diabetes, mitogenic, immunomodulatory, and anti-obesity properties [2,7,25].

Extracts enriched by yeast-derived peptides can be obtained through several techniques, including natural autolysis when proteolytic activity is obtained by the activation of endogenous enzymes at the end of yeast growth, although chemical or exogenous enzyme hydrolysis can also be employed [2,25]. Herein, commercial baker’s yeast biomass was successfully used to prepare a low mass, peptide-rich extract through autolysis induced by mild thermal treatment to favor proteolytic activity by peptidases, followed by ultracentrifugation in order to restrict peptide molecular masses under 10 kDa. The yeast extracts mainly composed of <10 kDa peptides inhibited clinical and foodborne bacteria, although in most of the cases at high concentrations, which corroborated previous studies reported by Fakruddin et al. [26] and Al-Sahlany et al. [27], who obtained antibacterial peptides after thermal treatment, although a non-*S. cerevisiae* baker lineage was employed in the former study. The estimated IC_50_ indicates that the FPLC fractionation of the filtered <10 kDa peptide extract into six peptide pool fractions may favor the improvement of antimicrobial activity that, alongside in silico predictions, suggest a latent potential of these peptides to inactivate bacteria. Considering that only fractions containing a peptide mixture were assayed concerning antimicrobial activity and cytotoxicity, further investigations should be performed with the individual sequences predicted herein as the best AMP candidates to confirm the effectiveness and/or cytotoxicity of each one. Upon validation, these data might indicate and combine a novel functional potential to *S. cerevisiae*-derived peptides, aiding in the control of infectious diseases that cannot be managed, or are poorly managed, by conventional antibiotics. Additionally, these AMP candidates could be used as a preventive treatment for immune-compromised individuals caused by different physiopathological conditions or associated with anti-neoplastic therapies avoiding opportunistic bacterial infections. Based on their nature, peptides from food matrices, like those obtained from baker’s yeast biomass, can also be included as an excipient in oral liquid formulations of pediatric medicines to preserve them and extend shelf-life while preventing excipient toxicity.

Although research concerning the applicability of *S. cerevisiae* peptides is in progress, little is known regarding the identification and physicochemical characterization of *Saccharomyces cerevisiae* AMPs, as most assessments are dedicated to the inhibition of bacteria and yeasts enrolled in industrial manufacturing, such as in wine-making conditions [7].

In the present study, the peptides that compose the filtered <10 kDa peptide extract were identified by nano-LC-MS/MS after fractionation by FPLC gel filtration chromatography, with 778 peptide hits distributed within, and shared by, the six obtained fractions. Together, the fractions contain 884 to 4344 Da peptides, presenting from 8 to 36 amino acid residues in length, and, considering that FPLC fractionation did not generate sharp and well separated peaks, it is expected that they share many peptide sequences. Except for 20 sequences, the peptides profiles, diversity, and amounts were distinct from the ones reported in the *S. cerevisiae* peptidome, comprised of 297 peptide sequences identified in the early-log phase, with some absent or decreased when yeasts were treated by proteasome inhibitors [28]. The GAFTGENSVDQIKDVGAK fragment encrypted in triosephosphate isomerase (*TPI1* gene) is a coincident sequence found herein and absent in the *S. cerevisiae* peptidome after proteasome inhibition by bortezomib, indicating that part of the peptides identified in the present study could be generated by protein turnover, a naturally-occurring process [28].

Other studies have reported in vitro and in vivo *S. cerevisiae* peptide health-promoting activities, including those attributed to brewer’s yeast or baker’s yeast, as mentioned previously [7]. Two saccharomycins, VSWYDNEYGYSTR and ISWYDNEYGYSAR, encrypted in glyceraldehyde-3-phosphate dehydrogenase (GAPDH) that converts glyceraldehyde-3-phosphate to 1, 3 bis-phosphoglycerate in the glycolytic pathway, encoded by the *TDH* gene (isoforms 1, 2, or 3), have been reported and characterized as a killer-like toxin naturally secreted by *S. cerevisiae*, with the former detected in the FLPC fractions, especially Fraction 4 (Table S2) [29,30,31,32]. Parts of GAPDH fragments are secreted, and the remaining fragments accumulate in the cell wall during wine fermentation at the end of the exponential phase growth due to the action of metacaspases and through direct inhibition and cell-to-cell contact, guaranteeing *S. cerevisiae* survival and dominance over non-*Saccharomyces* yeast and bacteria under wine-making conditions or other co-fermentation processes [31,33,34]. Coincidentally, some peptides identified in the fractions obtained herein are encrypted in non-Saccharomyces species such as *Kluyveromyces marxianus*, *Cyberlindnera fabianii*, *Candida glabrata*, *Schizosaccharomyces pombe*, *Candida albicans*, and *Zygosaccharomyces bailii* (Table S2, highlighted in red), which are known to perform alcoholic fermentation and are associated with starter cultures used in wine, beer, and baker’s yeasts production to confer organoleptic characteristics to the final product. This might be the reason why they were detected in the autolysate, and/or they may comprise the result of exogenous contamination [35]. Based on this, some GAPDH-peptides found herein may result from *S. cerevisiae* protective mechanisms activated during yeast biomass propagation to fight competition against microbial consortia.

The AMP candidates identified by mass spectrometry and described in Table 3 were not previously checked for antimicrobial activity according to BLAST searches at the antimicrobial peptide database CAMPR3 (http://www.camp.bicnirrh.res.in, accessed on 15 November 2021). None of the AMP candidates described herein exhibited a 100% identity with entire peptide sequences deposited in the database. A careful and detailed analysis must still be performed regarding AMP candidate partial similarity and physicochemical characteristics with previously checked AMPs. Although the VSWYDNEYGYSTR GAPDH fragment seems not to display the potential to inhibit foodborne and clinical bacteria based on the performed in silico screening, which did not classify this peptide within the AMP class (Table S2), this protein was proven a potential source for AMP generation. In fact, three other complementary fragments (Table 3) are among the best AMP candidates, while 10 other peptides were positive for one algorithm, indicating an antimicrobial character, although at a lower probability (Table S2). Even though most peptides present in F2 showed promising AMP potential according to in silico algorithms, when assayed in vitro the peptide pool did not exhibit the expected estimated antimicrobial potential. Coincidently, the F2 pool was the larger fraction with no defined peak, which justifies the highest number of peptides shared with the other fractions. It is possible that the high variety of peptides in the mixture interferes with, or prevails over, the performance of the best candidates predicted in F2. On the other hand, the peptide mixture in F1, the lowest peak, presented an estimated antimicrobial potential against most of the tested bacteria, while the in silico prediction did not reflect this result, as only 8 sequences were positively classified as notable AMP candidates, in contrast with the 55 sequences identified in F2. Moreover, the concentration of each individual peptide may also be a critical parameter influencing the antibacterial activity exerted by the peptide mixtures in all the fractions. The same is noted for fraction cytotoxicity, as the individual contribution of each peptide was not evaluated.

*Saccharomyces cerevisiae* acquired versatile characteristics throughout its evolutionary history 100 million years ago due to environmental pressure and, as a result of a series of critical events that include entire genome duplication, promoter rewiring, “Crabtree effect” emergence, the loss of the cis-regulatory motif, and gene mutations, leading to a unique fermentative ability even in the presence of oxygen and under unfavorable pH, temperature, osmolarity, and nutrient availability conditions or the presence of ethanol [1,2,4,36,37,38,39]. To cope with the adverse conditions imposed by fermentative environments, stress tolerance mechanisms are activated, triggering the up- and down-regulation of a set of 900 genes, mostly protein-encoding genes, involved in tolerance responses to guarantee *S. cerevisiae* survival [40,41,42,43,44,45]. This genome reprogramming consists of the expression of 5858 proteins, including both stress-related proteins and a variety of other protein classes constitutively expressed during yeast biomass propagation. All seem to be vital for cell physiology maintenance, such as structural proteins, protein machinery involved in protein biosynthesis necessary to survive abiotic stresses, proteins involved in protein folding, trafficking, and proteolysis, nuclear and mitochondrial proteins, and metabolic enzymes that make up part of this protein-rich yeast. Unsurprisingly, most of the peptides identified herein are encrypted in highly abundant proteins, such as those taking part in the glycolytic pathway, including the aforementioned GAPDH enzyme (*TDH1*/*2*/*3* gene), hexokinase isoenzyme 1 (*HXK1* gene), which is able to phosphorylate glucose, phosphoglycerate mutase (*GPM1* gene), that mediates the conversion of 3-phosphoglycerate to 2-phosphoglycerate, enolase I and II (*ENO1*/*2* gene), that catalyze the conversion of 2-phosphoglycerate to phosphoenolpyruvate, pyruvate kinase (*CDC19* gene), that converts phosphoenolpyruvate to pyruvate, 3-phosphoglycerate kinase (*PGK1* gene), a key enzyme in glycolysis and gluconeogenesis for ATP production, and triose phosphate isomerase (*TPI1* gene), which is involved in the breakdown of carbohydrates into pyruvate. Pyruvate decarboxylase (*PDC1* gene), which transforms pyruvate to acetaldehyde, alcohol dehydrogenase, and glucose-repressible alcohol dehydrogenase II (*ADH1*/*2* gene), which is required for the reduction of acetaldehyde to ethanol and the opposite, respectively, and aconitase (*ACO1* gene), which is required for the tricarboxylic acid (TCA) cycle, can both be considered as peptide precursors. Several other peptides are encrypted in abundant proteins enrolled in stress responses such as the 12 kDa heat shock protein (*HSP12* gene), heat shock proteins SSA1 and SSA2 (*SSA1* and *SSA2* genes), which are required for the ubiquitin-dependent degradation of short-lived proteins, and protein folding and elongation factors 1 and 2 (*EFT1*/*2* gene), which are active in protein biosynthesis.

Twenty-seven genes that encode many of these abundantly expressed proteins, such as *TDH1/2/3*, *ENO1/2*, *SSA1*, *PGK1*, *TEF1/2*, and *ADH1/2*, are the precursors of promising AMP candidates. Fraction 1 was shown to harbor eight promising AMP candidates, with six encrypted in the heat shock protein SSA1 (GGAPGGAAGGAPGGFPGGAPP, PGGAAGGAAGGAPGGFPGGAPP, PGGAAGGAAGGAPGGFPGG, GAPGGAAGGAPGGFPGG, GAPGGAAGGAPGGFPGGAPP, and GGAPGGAAGGAPGGFPGG), a member of the HSP70 family, one encrypted in phosphoglycerate kinase (*PGK1* gene) (IGDSIFDKA), and the other (SPGDGATFPK) in the chaperone-like FK506-binding protein 1. Potential candidates can also be encrypted in minor proteins, such as protein GRE1 (*GRE1* gene) and isomaltase (*IMA1* gene), indicating that, although they are not abundantly expressed, they are worthy of attention. In the same way, many peptides exhibiting low AMP potential when analyzed by in silico tools should be considered for further assessments by using synthetic sequences.

It is important to clarify that this is a preliminary study and that the observed results comprise the product of a mixture of peptides; further, the estimated cytotoxic concentration (IC_50_) and cytotoxicity (CC_50_), as well as the calculation of the selectivity index (SI), consist in a guiding character only in this study so that we could evaluate whether gel filtration fractionation would enrich the fractions with antimicrobial peptides or not in order to proceed with their identification by nano-LC/MS-MS. Furthermore, as mentioned previously, a detailed investigation should be carried out with the candidate peptide sequences chemically synthesized to point out each peptide contribution and determine not only their individual inhibitory (IC_50_) and cytotoxic (CC_50_) concentrations, but also the MIC. Only then can the antimicrobial potential and the low or null cytotoxicity of the AMP candidates identified in this study be confirmed in order to plan their future application in food products or drugs, considering their individual effective concentrations and not those estimated for the peptide pool, as was performed herein.

The IC_50_ and CC_50_ estimations, as well as the SI calculation associated with the in silico prediction, indicate that the F1 fraction contains a pool of promising peptides, with eight of them classified as candidate AMP peptides (Table 3). Thus, it seems that from all 58 identified candidates, future studies should be initiated with these eight peptides present in F1 and predicted as AMP candidates. Even so, the other candidate peptides cannot be discarded, and should also be evaluated separately if these eight candidates, isolated or combined with each other, do not show satisfactory antimicrobial potential against bacteria of clinical interest or foodborne pathogens.

## 4. Material and Methods

### 4.1. Organisms

Baker’s yeast cells from the Fleischmann brand were acquired in retail trade in the municipality of Rio de Janeiro (Latitude: S 22°54′13″, Longitude: W 43°12′35″), and used as a source of *Saccharomyces cerevisiae* ATCC 7754 [46].

Microorganisms of clinical importance and foodborne pathogens, kindly provided by the FIOCRUZ-INCQS cell bank and listed in Table 1, were used for the antimicrobial susceptibility assays.

A healthy human fibroblast lineage, HFF-1 (ATCC SCRC-1041), was purchased from Rio de Janeiro Cell Bank (BCRJ) and used for toxicological in vitro tests based on cell viability assays.

### 4.2. Autolysate Preparation

The peptide-rich autolysate extract was prepared according to Del Aguila et al. [47], with modifications. Briefly, powdered baker’s yeast (10.5 g) was homogenized in distilled water (50 mL) and the pH was adjusted to 6.0, followed by incubation in a water bath at 50 °C for 24 h under constant agitation to induce autolysis and proteolysis by endogenous enzymes. The resulting autolysate was centrifuged (Beckman Coulter, San Jose, CA, USA) at 8000× *g* for 10 min at room temperature and the proteolysis was stopped by incubating the supernatant at 90 °C for 10 min under constant agitation. Finally, the suspension was filtered through a 0.22 µm pore membrane (Merck Millipore Co., Darmstadt, Darmestádio, Germany), resulting in in the yeast autolysate extract, or autolysate.

### 4.3. Preparation of the Ultrafiltered Extract Containing <10 kDa Peptides

The autolysate was ultrafiltered through an Amicon ^®^ Ultra-15 (Merck Millipore Co.) with a 10 kDa cutoff membrane by centrifugation at 5000× *g* for 30 min [47]. Molecules with masses <10 kDa were collected and the filtered <10 kDa peptide extract was then stored at −20 °C until use.

### 4.4. Protein/Peptide Content Determination

The protein/peptide concentration of the samples was determined employing the Pierce BCA Protein Assay Kit (Thermo Fisher Scientific, Waltham, MA, USA) following the manufacturer’s instructions and previously described modifications [48,49,50,51].

### 4.5. Protein and Peptide Size Distribution Profile in the Autolysate and <10 kDa Filtered Peptides

The molecular masses of the proteins/peptides in both extracts were visualized on a 16% tricine-SDS-PAGE gel according to Schägger [52] using ultra-low range molecular weight markers (Invitrogen, Waltham, MA, USA). Peptide bands previously fixed with 5% glutaraldehyde were visualized under staining with 0.025% Brilliant Blue G.

The autolysate and <10 kDa filtered peptides extract were fractionated employing a LC-20A high-performance liquid chromatography (Shimadzu, Kyoto, Japan) using a ProSEC 300S 300 × 7.5 mm GPC/SEC gel filtration column (Agilent Technologies, Santa Clara, CA, USA) coupled to a photodiode array (PDA) detector model SPD-M30A (Shimadzu Corp., Kyoto, Japan). The chromatographic column was equilibrated with 0.05 M sodium phosphate buffer (Na_2_HPO_4_) pH 7, containing 0.15 M NaCl previously filtered through a 0.22 µm pore membrane (Merck Millipore Co.). Conalbumin (75 kDa), carbonic anhydrase (29 kDa), ribonuclease (13.7 kDa), and aprotinin (6.5 kDa) (GE Healthcare, Chicago, IL, USA) were used as molecular weight markers, dissolved in the mobile phase and filtered through a 0.45 µm pore membrane (Millipore). Fractionation was carried out at a flow rate of 1 mL/min at room temperature and absorbances were monitored at 280 nm and 215 nm.

### 4.6. Fractionation of the <10 kDa Filtered Peptides

The <10 kDa filtered peptides were fractionated by fast-performance liquid chromatography (FPLC) through a gel filtration column (Superdex-75/10 300GL) using the AKTApurifier 10 system (GE Healthcare). The chromatographic column previously equilibrated with 0.05 M sodium phosphate buffer pH 7.0 at a constant flow of 0.8 mL/min was loaded with 1 mL of the <10 kDa filtered peptide extract (14.78 mg), and absorbances were monitored at 280 and 215 nm while 1 mL fractions were collected. According to the chromatogram peaks, samples were pooled in six fractions (F1–F6).

### 4.7. Evaluation of Antimicrobial Activity in the Autolysate, <10 kDa Filtered Peptide Extract, and FPLC Gel Filtration Fractions

The antimicrobial activities of the autolysate and <10 kDa filtered peptides extracts, as well as F1–F6 FPLC peptide fractions, were assayed against foodborne pathogens and bacteria of clinical interest through the microdilution method to estimate the 50% inhibitory concentration (IC_50_) based on the Clinical & Laboratory Standards Institute (CLSI) recommendations, with adaptations [53]. The 50% inhibitory concentration (IC_50_) was predicted from inhibition curves constructed with increasing concentrations of crude extracts or F1–F6 samples using resazurin dye as an indicator to identify viable cells. As antimicrobial peptides were poorly represented in those complex fractions, the IC_50_ was used as an alternative for MIC (minimum inhibitory concentration) determination, to circumvent samples that did not reach 100% of growth inhibition at the tested concentrations, as recommended by the CLSI. Ten representative IC_50_ curves from a set of over 60 curves are represented in Figure S2.

Microorganisms were inoculated in Mueller Hinton Broth (MHB) containing 2.0 g/L meat extract, 17.5 g/L casamino acid, and 1.5 g/L starch (KASVI, PR, BR) and incubated at 37 °C for 18 h. Subsequently, a bacterial suspension containing 10^8^ cells was prepared according to the McFarland 0.5 scale followed by a serial 10-fold dilution in MHB. Autolysate, <10 kDa filtered peptide extract, and FLPC gel filtration fractions (F1–F6) aliquots were serially diluted 2-fold (at an initial concentration of 8370.45 µg/mL, 7393.41 µg/mL, 3.68 µg/mL, 376.09 µg/mL, 31.68 µg/mL, 13.13 µg/mL, 19.65 µg/mL, and 12.54 µg/mL, respectively) and mixed with the bacterial suspension at a final concentration of 10^7^ cells/mL. Microplates were incubated at 37 °C for 18 h under constant agitation and cell viability was then assessed by adding 30 µL of 0.02% resazurin, as described by McMillian et al. [54], followed by incubation at 37 °C for an additional 2 h. Fluorescence intensity was determined using a 2030 Multilabel Reader VICTOR ™ X4 microplate reader (PerkinElmer, Waltham, MA, USA) at 530 nm (excitation) and 590 nm (emission). Positive controls for the antimicrobial effects were comprised of chloramphenicol, cephalexin, and vancomycin (Sigma-Aldrich Co., San Luis, MO, USA), but only the former was reported herein.

The GraphPad prism version 9 software (GraphPad Software Inc., San Diego, CA, USA) was used to plot inhibition percentages of each sample concentration dilution (on a Log_10_ scale) and construct the inhibitory curves to predict the IC_50_ for each tested sample. Ten representative curves from a total of over 60 curves are shown in Figure S2.

### 4.8. Investigation of In Vitro Toxicity against Human Cell Lineages

The toxicological potential of both extracts and the collected fractions (F1–F6) were investigated according to Corrêa et al. [55] against a healthy human fibroblast lineage HFF-1 (ATCC SCRC-1041). Healthy human fibroblasts (5 × 10^5^ cells/mL) were seeded in 96-well microplates in high-glucose Dulbecco’s modified Eagle medium/Nutrient Mixture F-12 (DMEM/F-12, Ref# 11330-032) (Gibco, Gaithersburg, MD, USA), and supplemented with 10% fetal calf serum (FCS), respectively. To allow cell attachment, microplates were incubated for 24 h at 37 °C in a humidified atmosphere containing 5% CO_2_. The samples (autolysate, filtered <10 kDa peptide extract, and gel filtration fractions (F1–F6) were serially diluted 2-fold (at an initial concentration of 8370.45 µg/mL, 7393.41 µg/mL, 3.68 µg/mL, 376.09 µg/mL, 31.68 µg/mL, 13.13 µg/mL, 19.65 µg/mL, and 12.54 µg/mL, respectively) and added to the semi-confluent cell monolayer, followed by incubation for an additional 24 h. Cell viability was assessed by adding 0.02% resazurin (Sigma-Aldrich Co.) according to McMillian, Li, Parker, Patel, Zhong, Gunnett, Powers and Johnson [54], and fluorescence intensity was determined after 4 h of incubation employing a Victor ™ X microplate reader (PerkinElmer Inc.) at excitation and emission wavelengths of 530 and 590 nm, respectively.

The GraphPad prism version 9 software (GraphPad Software Inc.) was used to plot inhibition percentages of each sample concentration dilution (on a Log_10_ scale) and construct the inhibitory curves to predict the cytotoxic concentration (CC_50_) (Figure S3) for each tested sample. The selective index (SI) was calculated as CC_50_/IC_50_.

### 4.9. Nano-Liquid Chromatography and Mass Spectrometry (Nano-LC-MS/MS)

The peptides in each fraction (F1–F6) were identified by nano-LC-MS/MS according to Freitas et al. [56]. Nano-LC-MS/MS analyses were performed in triplicate and peptide chromatography separation was performed using a nano-LC Proxeon EASY-nLCII (Thermo Scientific) coupled to a Quadrupole-Orbitrap Q-Exactive Plus mass spectrometer (Thermo Scientific). Briefly, cleaned peptides were loaded onto a ReproSil-Pur C18-AQ 5µm pre-column (2 cm length, 200 µm inner diameter, packed in-house with resin) and fractionated using a ReproSil-Pur C18-AQ 3 µm column (15 cm length, 75 µm inner diameter, packed in-house with resin). A 5–50% mobile phase [water (solution A) and acetonitrile (solution B) gradient was applied for 60 min at flow rate of 300 nL/min (Time(min)/min/%solution B. Curve—1. Initial/2% B, 2. 5 min/10% B, 3. 30 min/30% B, 4. 60 min/50% B), at a flow rate of 300 nL/min]. The temperature was maintained at 35 °C and the no lock mass was used, delivered by an auxiliary pump at a flow rate of 200 nL/min. The peptide ionization conditions included a source temperature of 80 °C, a capillary voltage of 2600 V, positive polarity and a sample cone voltage of 35 V. Mass spectra of precursor peptides were acquired by a quadrupole mass analyzer (Full MS acquisition) at 70,000 of resolution, AGC target of 3 × 10^6^, maximum IT of 100 ms, range of 375–2000 m/z, and spectra were integrated over 1 s of scanning and with 0.1 s interscan intervals. The MS/MS mass spectra were acquired by an Orbitrap analyzer using dd-MS2 acquisition at 200 to 2000 m/z range by the twenty most intense ions (top N = 20) at 17,500 resolution, AGC target of 1 × 10^6^, maximum IT of 20 ms, and fixed first mass of 110 m/z.

### 4.10. Data Analysis and Protein Identification

The raw data obtained by Full-Scan-dd-MS^2^ acquisition were processed and analyzed using the Peaks X+ Pro software, version 10.6 20201221 (Bioinformatic Solutions, Waterloo, ON, Canada), matched against the *Saccharomyces cerevisiae* UNIPROTDB protein database (accessed on May 2021). The search parameters were set as follows: two missed cleavages, carbamidomethyl I as a fixed modification, oxidation (M) as a variable modification, 0.5 MS tolerance, 0.1 MS/MS tolerance, +2 +3 +4 or more charges, and False Discovery Rate (<1). All identifications were manually checked and those considered valid should comprise peptides with at least seven amino acid residues sequenced consecutively in the series y or b or in a complementary form. All peptides that did not meet this inclusion criterion were discarded.

L and I are isobars and cannot be differentiated using CID as the dissociation mechanism. When this mass difference is checked in the spectrum, the symbol X or Lxx must be used (L/I is another applied notation), according to Hunt et al. [57]. UNIPROT protein databases are basically divided into manually revised and unreviewed sequences and do not discriminate between annotated protein sequences by Hunt’s nomenclature or other alternatives for the discrimination of isobaric residues. Some of the amino acid sequences of the peptides described in this study belong to the revised and unrevised sequences set in UNIPROT DB. This information should be considered in future validation studies when using synthetic peptides.

### 4.11. In Silico Screening for Antimicrobial Peptide Candidates

Peptides identified by nano-LC-MS/MS in FPLC fractions were selected from an initial list of sequences, based on three occurrences in the triplicates and a cutoff point from −6 to 6 ppm. An in silico prediction was then performed using four algorithms freely available at the CAMPR3 website (http://www.camp.bicnirrh.res.in/prediction.php, accessed on 5 August 2021), including support vector machines (SVM), random analysis of artificial neural networks (RNA), and discriminant (RF and DA) algorithms to determine the probability of each peptide to exhibit antimicrobial activity [58]. Peptide sequences were classified as best AMP candidates if positive results were obtained for at least three algorithms, and good candidates if positive for two algorithms.

### 4.12. Statistical Analyses

All experiments were performed in triplicate and multiple comparison analyses were carried out using the ANOVA test followed by Tukey’s post hoc test [59]. Data were considered significant when *p* < 0.05, as determined by the GraphPad Prism version 9 software (GraphPad Software Inc.).

## 5. Conclusions

Food-grade *S. cerevisiae*-derived peptide pools with promising estimated antimicrobial potential were obtained from baker’s yeast biomass, a low-cost and non-seasonal food matrix, employing a green technology. Sequencing and in silico peptides prediction in <10 kDa peptide fractions revealed the presence of AMP candidate sequences that might harbor the potential to become the next generation of antimicrobials. Stress-related and metabolic proteins, abundantly expressed during baker’s yeast biomass manufacturing, are the main peptide fragment precursors composing the <10 kDa peptide fractions and might be involved in the observed antimicrobial potential. As the antimicrobial activity described herein was an estimated result from a complex peptide mixture, the potential AMP candidates previously identified by nano-LC MS/MS must be individually analyzed to validate their functionality. Experimental validation should be comprised of a screening of synthetic sequences against foodborne pathogens or clinical bacteria. The therapeutic efficacy of *S. cerevisiae* peptides can be customized by combining two or more oligopeptide sequences but keeping the non-toxicity status. Yeast antimicrobial peptides can also be joined to nanocarriers/nanomaterials, which is another approach to increase the antimicrobial effectiveness by protecting peptide molecules while guaranteeing effective concentrations for longer periods. Finally, and maybe the most relevant aspect, the broad application of these antimicrobial peptides emerges as a strategy to reduce or decelerate the development of bacterial resistance.

## Figures and Tables

**Table 1:** Estimated inhibitory potential (IC_50_) of the autolysate, filtered <10 kDa peptide extract, FPLC gel filtration fractions, and antibiotics.

Microorganism	Gram Staining	Yeast Extracts (μg/mL)		FPLC Gel Filtration Fractions (μg/mL)						Antibiotic Reference (μg/mL)
		Autolysate	<10 kDa Filtered Peptides	F1	F2	F3	F4	F5	F6	CPL
*Acinetobacter* genomospecies 3 *	negative	9088	8484	88.34 ****	-	-	-	-	-	29.84
*Aeromonas hydrophila* ATCC 7966	negative	994.2 **	1939	10.04 ****	436.3 ****	109.1 ****	37.25 ****	-	-	-
*Bacillus cereus* ATCC 11778	positive	428.5	547.9	66.96 **	-	665.3	78.76 *	508.8	-	0.57
*Escherichia coli* CDC EDL-933	negative	7468	6439	103.4 ****	2424	-	48.56 ****	6816	-	-
*Escherichia coli* CDC O55	negative	6717	9939	124.5 ****	7680	-	435.3 ***	578.9 ***	-	0.64
*Escherichia coli* DH5α	negative	3549	5527	453.4 ****	2840	-	96.32 ****	206.5 ****	-	-
*Salmonella enterica* ATCC 12325	negative	5833	5590	164.0 ****	13676	-	1117 **	5087	-	0.68
*Shigella flexneri* ATCC 12022	negative	2884	4014	-	35,516 **	-	-	-	-	0.01
*Shigella sonnei* ATCC 25931	negative	2091	2970	49.47 ****	4641	-	-	-	-	0.99
*Staphylococcus aureus* ATCC 14458	positive	1696	2177	-	1190	-	452.8 *	331.4 **	333.4 **	36.73

* Sludge isolate, Pinhati et al. [15]; ATCC, American Type Culture Collection; CDC, Center for Disease Control (USA). The IC_50_ was estimated with the aid of GraphPad Prism v.9 software from the inhibitory growth curves of each species cultivated under different sample concentrations. Growth inactivation assays were performed in triplicate. Ten IC_50_ representative curves, from a set of 60, are included in Figure S2. (-) Not Inhibited; CPL, chloramphenicol; * *p* < 0.05, ** *p* < 0.01, *** *p* < 0.001, and **** *p* < 0.0001 indicate a significant difference compared to the <10 kDa filtered peptides extract. Multiple comparison analyses were carried out using the one-way ANOVA test followed by Tukey’s post hoc test.

**Table 2:** Estimated cytotoxic concentrations (CC_50_) and selective indices (SI) of the yeast extracts and FPLC gel filtration fractions against a healthy human fibroblast cell lineage (HFF-1).

Estimated Indices	Cell Lineage	Yeast Extracts		FPLC Gel Filtration Fractions					
		Autolysate	<10 kDa Filtered Peptides	F1	F2	F3	F4	F5	F6
**CC_50_** (μg/mL)	Human HFF-1 lineage ATCC SCRC-1041	1464 *	3256	4545 ****	4642 ****	3911 ****	1622 ****	2427 ****	1549 ****
**SI**	*Acinetobacter* genomospecies 3 *	0.16	0.38	**51.45**	**-**	**-**	**-**	**-**	**-**
	*Aeromonas hydrophila* ATCC 7966	1.47	1.68	**452.69**	**10.64**	**35.85**	**43.54**	**-**	**-**
	*Bacillus cereus* ATCC 11778	3.42	5.94	**67.88**	-	5.88	**20.59**	4.77	**-**
	*Escherichia coli* CDC EDL-933	0.20	0.51	**43.96**	1.92	**-**	**33.40**	0.36	**-**
	*Escherichia coli* CDC O55	0.22	0.33	**36.51**	0.60	**-**	3.73	4.19	**-**
	*Escherichia coli* DH5α	0.41	0.59	**10.02**	1.63	**-**	**16.84**	**11.75**	**-**
	*Salmonella enterica* ATCC 12325	0.25	0.58	**27.71**	0.34	**-**	1.45	0.48	**-**
	*Shigella flexneri* ATCC 12022	0.51	0.81	**-**	0.13	**-**	**-**	**-**	**-**
	*Shigella sonnei* ATCC 25931	0.70	1.10	**91.87**	1.00	**-**	**-**	**-**	**-**
	*Staphylococcus aureus* ATCC 14458	0.86	1.50	**-**	3.90	**-**	3.58	7.32	4.65

* Sludge isolate [15]; ATCC, American Type Culture Collection; CDC, Center for Disease Control (USA). The CC_50_ was estimated with the aid of GraphPad Prism v.9 software from the inhibitory growth curves of the HFF-1 cells, performed in triplicate, cultivated under different sample concentrations as shown in Figure S3. SI, the selective index, was calculated as the CC_50_/IC_50_ ratio. (-) Not Inhibited. The most promising SI values (≥10) as reported by [16] are displayed in bold. * *p* < 0.05 and **** *p* < 0.0001 indicate differences compared to <10 kDa filtered peptides. Multiple comparison analyses were carried out using the one-way ANOVA test followed by Tukey’s post hoc test.

**Table 3:** Potential AMP candidates screened by an in silico investigation using four independent algorithms.

Peptide Sequence	Molecular Mass	M/Z	Protein	Gene	Entry Name	F1	F2	F3	F4	F5	F6	AMPs Prediction
GENVKGWKIGDYAGIK	1733.91	867.9674	Alcohol dehydrogenase ½	ADH1/2	ADH1_YEAST/ADH2_YEAST		x					++++
IDNLLDKVDSIIIGGG	1640.8984	821.4605	Phosphoglycerate kinase	PGK1	PGK_YEAST		x					++++
IDNLLDKVDSIIIGGGM	1771.939	886.9802	Phosphoglycerate kinase	PGK1	PGK_YEAST		x					++++
IPAPRGSGIVASPA	1291.7247	646.8714	40S ribosomal protein S2	RPS2	RS2_YEAST		x					++++
GLIKSPIKV	953.6273	477.8238	Alcohol dehydrogenase	SCRG_01319	B3LIX8_YEAS1		x					++++
GAPGGFPGGAPP	980.4715	491.2457	Heat shock protein SSA1	SSA1	HSP71_YEAST		x					++++
KIGGIGTVPVGRVETGVIKPG	2033.1997	1017.6146	Elongation ator 1-alpha	TEF1/2	EF1A_YEAST		x					++++
VDIGKNEGATLITGGERLGSK	2114.1331	705.7211	Potassium-activated aldehyde dehydrogenase. Mitochondrial. EC 1.2.1.5	ALD4	ALDH4_YEAST		x					+++−
GGTLNPGLAPAPVHKF	1574.8568	788.4407	Ammonia transport outward protein 2/Accumulation of dyads protein 2	ATO2/ADY2	ATO2_YEAST/ADY2_YEAST		x					+−++
LISLDGTANKSKLGAN	1600.8784	534.6364	Enolase 1	ENO1	ENO1_YEAST		x					+−++
NLLDKVDSIIIGGG	1412.7875	707.4042	Phosphoglycerate kinase	PGK1	PGK_YEAST		x					+−++
NLLDKVDSIIIGGGM	1543.828	772.9217	Phosphoglycerate kinase	PGK1	PGK_YEAST		x					+−++
VGKVLPELQGKL	1279.7864	640.904	Glyceraldehyde-3-Phosphate dehydrogenase 1/2/3	TDH1/2/3	G3P1_YEAST/G3P2_YEAST/G3P3_YEAST		x					+−++
GNIVDVPVGPGLLGRV	1560.8987	781.4649	ATP synthase subunit alpha, mitochondrial	ATP1	ATPA_YEAST		x					−+++
GAPGGAAGGAAGGAPGGFPGGAPPAPE	2071.9709	1037.0002	XXYS1_4_G0051300.mRNA.1.CDS.1	PACBIOSEQ_LOCUS72	A0A7I9C8D2_YEASX		x					−+++
PGGAAGGAAGGAPGGFPGGAPPAPE	1943.9125	972.9683	XXYS1_4_G0051300.mRNA.1.CDS.1	PACBIOSEQ_LOCUS72	A0A7I9C8D2_YEASX		x					−+++
GAPGGAAGGAPGGFPGGAPPAPE	1815.8539	908.9403	Heat shock protein SSA1	SSA1	HSP71_YEAST		x	x	x			−+++
**GGAPGGAAGGAPGGFPGGAPP**	1575.7429	788.8842	Heat shock protein SSA1	SSA1	HSP71_YEAST	x	x					−+++
GGAPGGAAGGAPGGFPGGAPPAPE	1872.8754	937.4507	Heat shock protein SSA1	SSA1	HSP71_YEAST		x					−+++
DWRGGRTASGNIIPSSTGAAK	2101.0664	701.3662	Glyceraldehyde-3-Phosphate dehydrogenase 1/2/3	TDH1/2/3	G3P1_YEAST/G3P2_YEAST/G3P3_YEAST		x					−+++
EHTPRHHQYGSDEGEQDYHDDEQGEEQAGKQ	3635.4805	728.1075	Protein HBT1	HBT1	HBT1_YEAST		x					++−−
IVDVPVGPGLLGRV	1389.8344	695.9295	ATP synthase subunit alpha, mitochondrial	ATP1	ATPA_YEAST		x					−++−
IDEIDSIAPK	1099.576	550.7982	Cell division control protein 48	CDC48	CDC48_YEAST		x					−++−
**SPGDGATFPK**	975.4661	488.7423	FK506-binding protein 1	FPR1	FKBP_YEAST	x	x					−++−
IDDVDSIIKN	1130.5819	566.3014	Homocitrate synthase, cytosolic isozyme	LYS20	HOSC_YEAST		x					−++−
IDDVDSIIK	1016.5389	509.2791	Homocitrate synthase, cytosolic isozyme/Homocitrate synthase, mitochondrial	LYS20/21	HOSC_YEAST/HOSM_YEAST		x					−++−
LPANLVDLNVPAKL	1475.8711	738.9482	Pyruvate decarboxylase isozyme ½	PDC1/5	PDC1_YEAST/PDC5_YEAST		x					−++−
VDLNVPAKL	967.5702	484.7956	Pyruvate decarboxylase isozyme ½	PDC1/5	PDC1_YEAST/PDC5_YEAST		x					−++−
GIGTVPVGRVETGVIKPG	1734.9991	868.51	Elongation factor 1-alpha	TEF1 /2	EF1A_YEAST		x					−++−
IGTVPVGRVETGVIKPG	1677.9777	840.0009	Elongation factor 1-alpha	TEF1 /2	EF1A_YEAST		x					−++−
IIAGGVGEFEAGISKDGQTREHA	2341.1663	586.3022	Elongation factor 1-alpha	TEF1 /2	EF1A_YEAST		x					−++−
GIGTVPVGRV	953.5658	477.7927	Elongation factor 1-alpha, EF-1-alpha	TEF1/2	EF1A_YEAST		x					−++−
IGGIGTVPVGRVE	1252.7139	627.3672	Elongation factor 1-alpha	TEF1/2	EF1A_YEAST		x	x	x			−++−
VPIGRGQRELIIGDR	1677.9637	560.3348	ATP synthase subunit alpha, mitochondrial	ATP1	ATPA_YEAST		x					−−++
GGAPGGAAGGAAGGAPGGFPGGAPPAPE	2128.9924	1065.5073	XXYS1_4_G0051300.mRNA.1.CDS.1	PACBIOSEQ_LOCUS72	A0A7I9C8D2_YEASX		x					−−++
**PGGAAGGAAGGAPGGFPGG**	1381.6375	691.8289	XXYS1_4_G0051300.mRNA.1.CDS.1	PACBIOSEQ_LOCUS72	A0A7I9C8D2 (A0A7I9C8D2_YEASX)	x						−−++
**PGGAAGGAAGGAPGGFPGGAPP**	1646.78	824.4052	XXYS1_4_G0051300.mRNA.1.CDS.1	PACBIOSEQ_LOCUS72	A0A7I9C8D2 (A0A7I9C8D2_YEASX)	x	x					−−++
PGGPGGAGGAGGFPGGAGG	1353.6061	677.811	Protein SIS1	SIS1	SIS1_YEAST		x					−−++
**GAPGGAAGGAPGGFPGG**	1253.5789	627.8004	Heat shock protein SSA1	SSA1	HSP71_YEAST	x						−−++
**GAPGGAAGGAPGGFPGGAPP**	1575.7429	760.3733	Heat shock protein SSA1	SSA1	HSP71_YEAST	x	x	x				−−++
**GGAPGGAAGGAPGGFPGG**	1310.6003	656.3116	Heat shock protein SSA1	SSA1	HSP71_YEAST	x						−−++
STGAAKAVGKVLPELQGK	1753.0098	585.3472	Glyceraldehyde-3-phosphate dehydrogenase 1/2/3	TDH1/2/3	G3P1_YEAST/G3P2_YEAST/G3P3_YEAST		x					−−++
IGGIGTVPVGRV	1123.6713	562.8463	Elongation factor 1-alpha	TEF1/2	EF1A_YEAST		x	x				−−++
GAPAPPPPPPPPALGGSAPKP	1869.0148	935.5192	Verprolin	VRP1	VRP1_YEAST		x					−−++
GGFGGPGGPGGQGFGRQGPQG	1827.84	914.9275	Uncharacterized protein YNL208W	YNL208W, N1338	YNU8_YEAST		x					−−++
EVEKEVPIPEEEKKDEEKKDEEKKDEDDKKPKLE	4138.0688	690.6852	ATP-dependent molecular chaperone HSP82	HSP82	HSP82_YEAST		x					−+−+
APGGAAGGAPGGFPGGAPPAPE	1758.8324	880.4313	Heat shock protein SSA1	SSA1	HSP71_YEAST		x					−+−+
WKIGDYAGIK	1149.6182	575.8202	Alcohol dehydrogenase ½	ADH1/2	ADH1_YEAST/ADH2_YEAST		x					+−+−
APPLPRAPPVPP	1207.7076	604.8652	Myosin tail region-interacting protein MTI1	BBC1	BBC1_YEAST		x					+−+−
LLSLDGTANKSKLGAN	1600.8784	534.6364	Enolase 2	ENO2	ENO2_YEAST		x					+−+−
LDQEPDAGLGNGGLGRL	1680.843	841.4357	Glycogen phosphorylase	GPH1	PHSG_YEAST		x					+−+−
VLDQEPDAGLGNGGLGRL	1773.8685	890.9691	Glycogen phosphorylase	GPH1	PHSG_YEAST		x					+−+−
**IGDSIFDKA**	964.4865	483.253	Phosphoglycerate kinase	PGK1	PGK_YEAST	x	x		x			+−+−
FKNPNSDKSKWLTGPQ	1845.9373	923.9781	Enolase 1	ENO1	ENO1_YEAST		x					+−−+
GSKADPYGEENQGNFPQRQQPQ	2474.1211	1238.0784	Protein GRE1	GRE1	GRE1_YEAST		x					+−−+
NNYNAIKEEHGENSEEMKKF	2410.0859	804.3777	Oligo-1,6-Glucosidase IMA1	IMA1	MALX3_YEAST		x					+−−+
PPPVFNKPPTGPPP	1440.7765	721.3985	Protein transport protein SEC31	SEC31	SEC31_YEAST		x					+−−+
SPPPVFNKPPTGPPP	1527.8085	764.9156	Protein transport protein SEC31	SEC31	SEC31_YEAST		x					+−−+

M/Z—mass/charge ratio; a positive (+) sign indicates that the peptide tested positive for the algorithm, and a negative (−) sign indicates that the peptide tested negative. (x) sign indicates the presence of the peptide in all three replicates of the fraction. Peptides in bold represent the AMP candidates found in F1, the most effective antimicrobial fraction.

## Data Availability

The data are contained within the article.

## Supplementary Materials

The following are available online at https://www.mdpi.com/article/10.3390/ph15020163/s1. Figure S1: HPLC fractionation of molecular weight markers, Figure S2: Representative dose-dependent curves for the IC_50_ estimation of FPLC fractions—F1, F2, F4, and F5, Figure S3: Inhibition curves of yeast extracts and FPLC gel filtration fractions assayed against healthy human HFF-1 fibroblasts. Table S1: Fractionation table of FPLC gel filtration fractions, Table S2: Peptide hits identified in the FPLC gel filtration fractions obtained from <10 kDa filtered peptide extract.
