# Supplementary material for: Identification of Antibacterial Peptide Candidates Encrypted in Stress-Related and Metabolic Saccharomyces cerevisiae Proteins
Source: Pharmaceuticals (Basel). 2022 Jan 28;15(2):163. doi: 10.3390/ph15020163 (PMC8877035; doi:10.3390/ph15020163)
Supplement: Supplementary file 1 [file pharmaceuticals-15-00163-s001.zip › pharmaceuticals-1568877-supplementary/Figure S1_with legend.pdf]

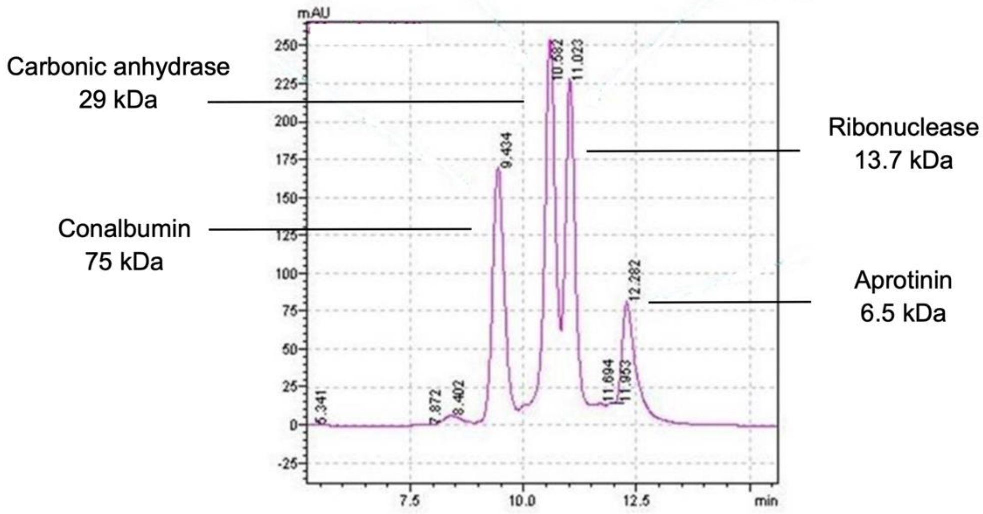

Figure S1. HPLC fractionation of molecular weight markers. Conalbumin (75 kDa), carbonic anhydrase (29 kDa), ribonuclease (13.7 kDa) and aprotinin (6.5 kDa) were fractionated through an HPLC system coupled to a gel filtration ProSec300S column equilibrated with 0.05 M sodium phosphate buffer pH 7, and eluted in the same buffer containing 0.15 M NaCl at a flow rate of 1 mL/min. Absorbance was monitored at 215 nm.
