# Supplementary material for: Identification of Antibacterial Peptide Candidates Encrypted in Stress-Related and Metabolic Saccharomyces cerevisiae Proteins
Source: Pharmaceuticals (Basel). 2022 Jan 28;15(2):163. doi: 10.3390/ph15020163 (PMC8877035; doi:10.3390/ph15020163)
Supplement: Supplementary file 1 [file pharmaceuticals-15-00163-s001.zip › pharmaceuticals-1568877-supplementary/Figure S2_with legend.pdf]

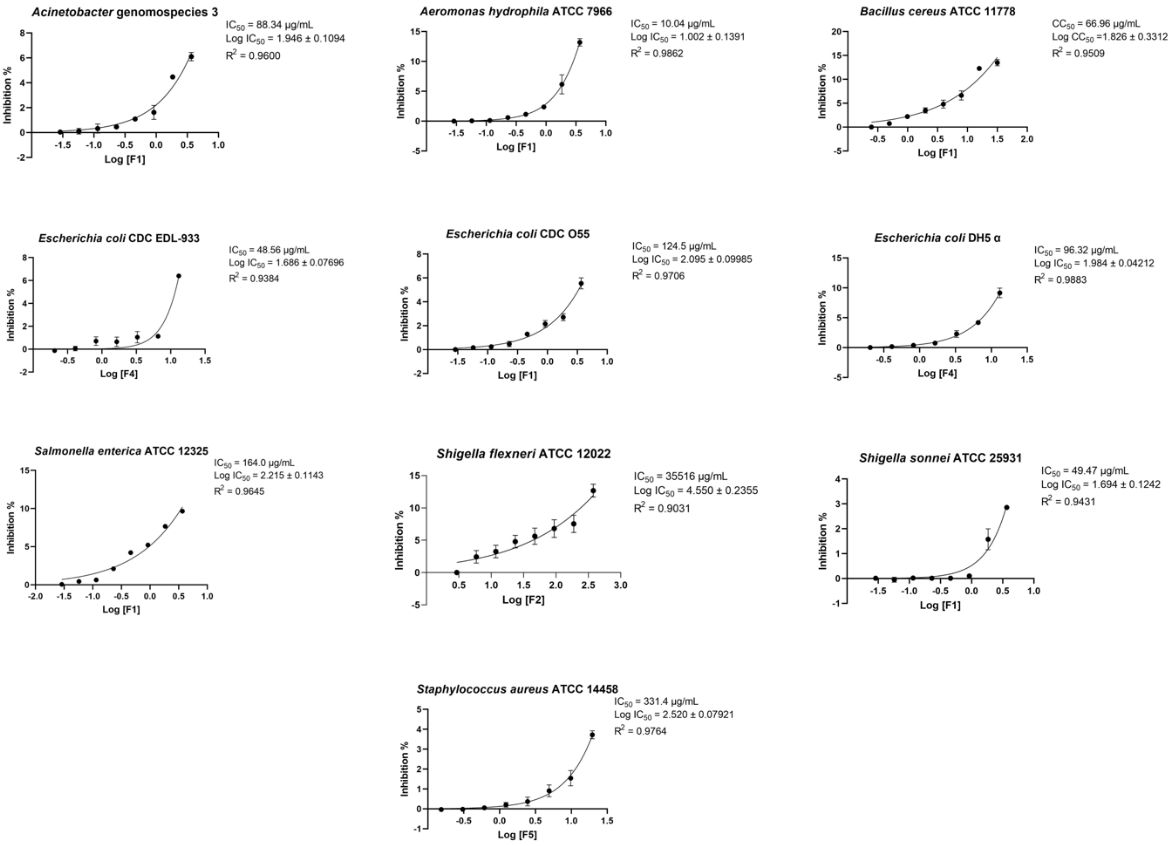

Figure S2. Representative dose-dependent curves for IC_{50} estimations of FPLC fractions F1, F2, F4 and F5. The autolysate, filtered <10kDa peptide extract and gel filtration fractions were 2-fold serially diluted and added to the bacterial culture (10^7 cells/mL) for 18 h at 37 °C under constant agitation. Cell viability was assessed by adding 0.02% resazurin and fluorescence intensity determined after 2 h incubation at excitation and emission wavelengths of 530 and 590 nm, respectively, generating nearly 60 curves. The 50% inhibitory concentrations (IC_{50}) were estimated from the inhibition curves using the GraphPad prism version 9 on a log scale. Results are expressed as the means for triplicates for the IC_{50} and means ±SE for $LogCC_{50}$.
