# Supplementary material for: Identification of Antibacterial Peptide Candidates Encrypted in Stress-Related and Metabolic Saccharomyces cerevisiae Proteins
Source: Pharmaceuticals (Basel). 2022 Jan 28;15(2):163. doi: 10.3390/ph15020163 (PMC8877035; doi:10.3390/ph15020163)
Supplement: Supplementary file 1 [file pharmaceuticals-15-00163-s001.zip › pharmaceuticals-1568877-supplementary/Figure S3_with legend.pdf]

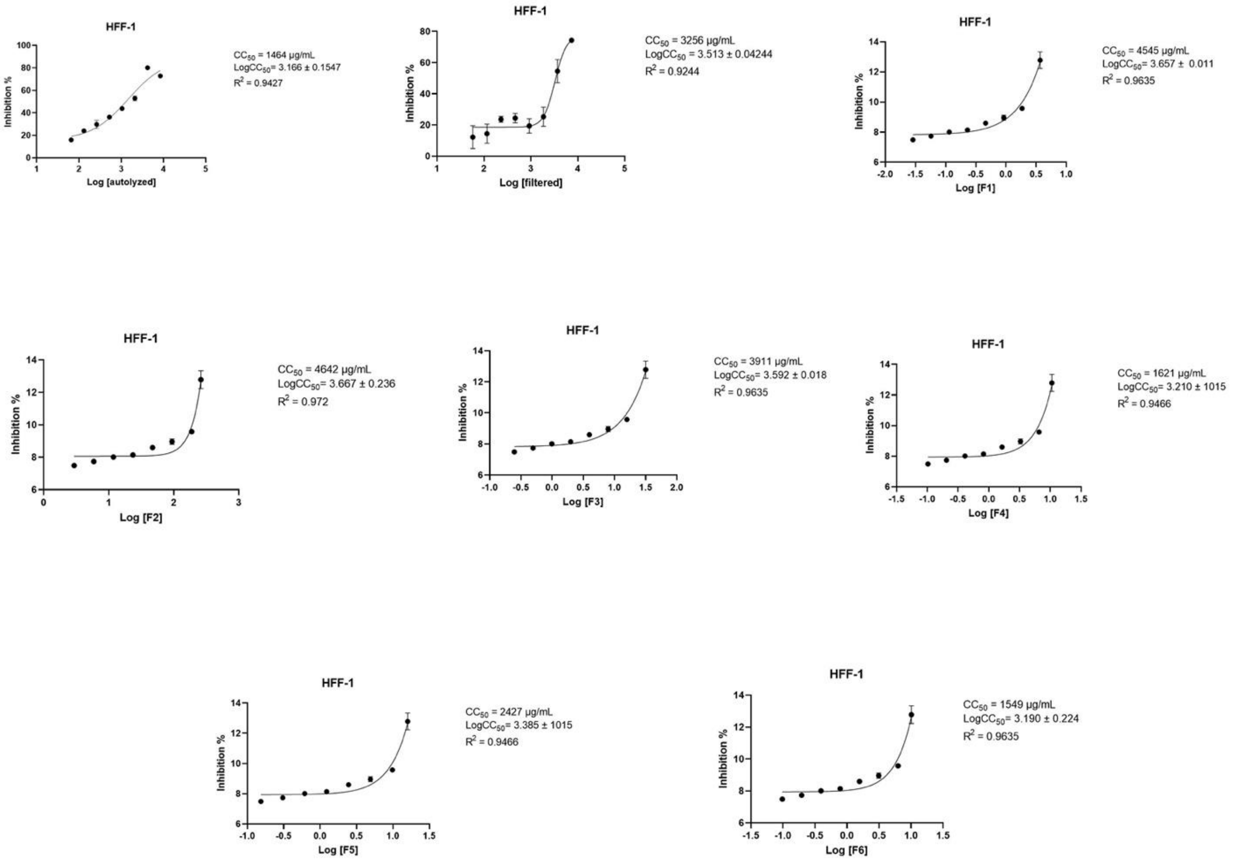

Figure S3. Inhibition curves of yeast extracts and FPLC gel filtration fractions assayed against healthy human HFF-1 fibroblasts. The autolysate, filtered <10kDa peptide extract and gel filtration fractions were 2-fold serially diluted and added to the semi-confluent cell monolayer followed by incubation for 24 h at 37 °C in a humidified atmosphere containing 5% CO_2. Cell viability was assessed by adding 0.02% resazurin and fluorescence intensity determined after 4 h incubation at excitation and emission wavelengths of 530 and 590 nm, respectively. The 50% cytotoxic concentration (CC_{50}) values were calculated from the inhibition curves using the GraphPad prism version 9 on a log scale. Results are expressed as means of a triplicate for the CC_{50} and means ±SE for $LogCC_{50}$.
