# Supplementary material for: Identification of Antibacterial Peptide Candidates Encrypted in Stress-Related and Metabolic Saccharomyces cerevisiae Proteins
Source: Pharmaceuticals (Basel). 2022 Jan 28;15(2):163. doi: 10.3390/ph15020163 (PMC8877035; doi:10.3390/ph15020163)
Supplement: Supplementary file 1 [file pharmaceuticals-15-00163-s001.zip › pharmaceuticals-1568877-supplementary/Table S1.pdf]

Supplementary file

Table S1. Fractionation table of the FPLC gel filtration fractions

Fractionation Steps	Fractions	Protein concentration (mg/mL)	Volume (mL)	Total protein (mg)	[a]Total activity (AU*)	[b]Specific Activity (AU/mg)	[c]Recovery (%)	[d]Fractionation fold
Aeromonas hydrophila ATCC 7966								
Ultrafiltration	<10 kDa filtered peptides	14.780	1	14.780	38.11	2.58	100	1
FPLC gel filtration	F1	0.007	3	0.022	11.00	498.01	29	193
	F2	0.752	19	14.291	163.78	11.46	430	4
	F3	0.064	6	0.382	17.50	45.83	46	18
	F4	0.026	6	0.158	21.16	134.23	56	52
Escherichia coli CDC EDL-933								
Ultrafiltration	<10 kDa filtered peptides	14.780	1	14.78	11.48	0.78	100	1
FPLC gel filtration	F1	0.00736	3	0.02	1.07	48.36	9	62
	F4	0.02627	6	0.16	16.23	102.97	141	133
Escherichia coli CDC O55								
Ultrafiltration	<10 kDa filtered peptides	14.780	1	14.78	7.44	0.50	100	1
FPLC gel filtration	F1	0.00736	3	0.02	0.89	40.16	12	80
	F4	0.02627	6	0.15762	1.81	11.49	24	23
	F5	0.03931	4	0.15724	1.36	8.64	18	17
Escherichia coli DH5α								
Ultrafiltration	<10 kDa filtered peptides	14.780	1	14.78	13.37	0.90	100	1
FPLC gel filtration	F1	0.00736	3	0.02	0.24	11.03	2	12
	F4	0.02627	6	0.15762	8.18	51.91	61	57
	F5	0.03931	4	0.15724	3.81	24.21	28	27
Acinetobacter genomospecies *3*								
Ultrafiltration	<10 kDa filtered peptides	14.780	1	14.78	8.71	0.59	100	1

FPLC gel filtration	F1	0.00736	3	0.02208	1.25	56.60	14	96
Shigella sonnei ATCC 25931								
Ultrafiltration	<10 kDa filtered peptides	14.780	1	14.78	24.88	1.68	100	1
FPLC gel filtration	F1	0.00736	3	0.02	2.23	101.07	9	60
Bacillus cereus ATCC 11778								
Ultrafiltration	<10 kDa filtered peptides	14.780	1	14.780	134.88	9.13	100	1
FPLC gel filtration	F1	0.007	3	0.022	1.65	74.67	1	8
	F3	0.064	6	0.382	2.87	7.51	2	1
	F4	0.026	6	0.158	10.01	63.48	7	7
	F5	0.039	4	0.157	1.54	9.83	1	1
Salmonella enterica ATCC 12325								
Ultrafiltration	<10 kDa filtered peptides	14.780	1	14.78	13.22	0.89	100	1
FPLC gel filtration	F1	0.00736	3	0.02208	0.67	30.49	5	34
Staphylococcus aureus ATCC 14458								
Ultrafiltration	<10 kDa filtered peptides	14.780	1	14.78	33.95	2.30	100	1
FPLC gel filtration	F4	0.02627	6	0.16	1.74	11.04	5	5
	F5	0.03931	4	0.16	2.37	15.09	7	7
	F6	0.02509	5	0.13	1.88	15.00	6	7

*One antimicrobial unit (AU) corresponds to the minimum concentration necessary to cause 50% growth inhibition (IC_{50}) in mg/mL adjusted to the final volume of the antimicrobial assay (200 μL).

[a] Total activity was calculated by the ratio between total protein and one AU, corresponding to the total AU present in the whole fraction.

[b] Specific activity (AU/mg) corresponds to the ratio between total activity (AU) and total protein (mg).

[c] The recovery yield (%) corresponds to the ratio between the total activity of the FPLC fraction and the <10 kDa filtered peptides, multiplied by 100.

[d] The fractionation fold corresponds to the ratio between the specific activity of FPLC fraction and the <10 kDa filtered fraction.

Fractions F1-F6 were obtained by FPLC gel filtration fractionation of the <10 kDa filtered peptide extract.
